# Supplement intake in half-marathon, (ultra-)marathon and 10-km runners – results from the NURMI study (Step 2)

**DOI:** 10.1186/s12970-021-00460-2

**Published:** 2021-09-27

**Authors:** Wirnitzer Katharina, Motevalli Mohamad, Tanous Derrick, Gregori Martina, Wirnitzer Gerold, Leitzmann Claus, Hill Lee, Rosemann Thomas, Knechtle Beat

**Affiliations:** 1 Department of Subject Didactics and Educational Research and Development, University College of Teacher Education Tyrol, Innsbruck, Austria; 2 Department of Sport Science, University of Innsbruck, Innsbruck, Austria; 3 Life and Health Science Cluster Tirol, Subcluster Health/Medicine/Psychology, Innsbruck, Austria; 4 Research Center Medical Humanities, Leopold-Franzens University of Innsbruck, Innsbruck, Austria; 5 Faculty of Physical Education and Sports Sciences, Ferdowsi University of Mashhad, Mashhad, Iran; 6 Department of Nutritional Sciences, University of Vienna, Vienna, Austria; 7 adventureV & change2V, Stans, Austria; 8 Institute of Nutrition, University of Gießen, Gießen, Germany; 9 Divison of Gastroenterology and Nutrition, Department of Pediatrics, McMaster University, Hamilton, Canada; 10 Institute of Primary Care, University of Zurich, Zurich, Switzerland; 11 Medbase St. Gallen Am Vadianplatz, St. Gallen, Switzerland

**Keywords:** Nutrition, Nutrients, Diet, Supplementation, Ergogenic aid, Long distance, Endurance, Running

## Abstract

The primary nutritional challenge facing endurance runners is meeting the nutrient requirements necessary to optimize the performance and recovery of prolonged training sessions. Supplement intake is a commonly used strategy by elite and recreational distance runners to meet nutritional recommendations. This study was conducted to investigate the patterns of supplement intake among different groups of distance runners and the potential association between supplement intake and sex, age, running and racing experiences.

In a cross-sectional design, from a total of 317 runners participating in this survey, 119 distance runners were involved in the final sample after data clearance, assigned into three groups of 10-km runners (n = 24), half-marathoners (*n* = 44), and (ultra-)marathoners (n = 51). Personal characteristics, training and racing experiences, as well as patterns of supplement intake, including type, frequency, and dosage, were evaluated by questionnaire. Food Frequency Questionnaire was implemented to assess macronutrient intake. ANOVA and logistic regression were used for statistical analysis.

While 50 % of total distance runners reported consuming supplements regularly, no differences between distance groups in consumption of carbohydrate/protein, mineral, or vitamin supplements were observed (p > 0.05). In addition, age, sex, running and racing experience showed no significant association with supplement intake (*p* > 0.05). Vitamin supplements had the highest intake rate in runners by 43 % compared to minerals (34 %) and carbohydrate/protein supplements (19 %).

The present findings provide a window into the targeted approaches of long-distance runners as well as their coaches and sport nutrition specialists when applying and suggesting sustainable nutritional strategies for training and competition.

**Trial registration**: ISRCTN73074080. Retrospectively registered 12th June 2015.

## Introduction

Running is one of the most popular sports, leisure activities, and health-promoting approaches, with growing popularity worldwide [1]. During the past two decades, there has been increasing participation in long-distance races – namely marathon and half-marathon events [2]. Therefore, these events represent an interesting and invaluable source of data to explore the various challenges that may be experienced among distance runners [3, 4]. It is well established that endurance runners are generally recommended to consume high-carbohydrate meals in the days leading up to a specific event, in conjunction with avoiding foods high in fat, protein, and fibre in pre-competition hours [5, 6]. Regardless of in-race nutritional strategies and the higher exercise-induced energy needs, endurance athletes may have different physiological requirements for macronutrients and micronutrients in optimizing their physiological adaptations during different preparation phases compared to other athletes [7]. Insufficient nutrient intake in endurance athletes may result in clinical and non-clinical deficiencies affecting both health and performance [8], particularly with long-term involvement in training and racing activities [9]. The nutrient requirements of endurance runners increase along with concomitant increases in intensity, frequency, and duration of running [10], thus providing a unique challenge of optimizing energy availability that shorter distance runners may not necessarily experience.

Considering the nutritional and energy demands associated with endurance training, athletes may utilize dietary supplements to support their performance during training and competition [11]. Dietary supplements were recently defined as products purposefully ingested to address nutritional, clinical, and performance goals, alongside a habitually consumed diet [12]. Supplement intake is a commonly used strategy among many professional and amateur athletes in order to meet the higher exercise-induced nutrient needs, match nutritional recommendations and/or enhance athletic performance [13]. Additionally, athletes may consume supplements for further reasons such as optimizing their adaptations, avoiding exercise- or diet-induced physical and physiological distresses, and speeding up recovery and rehabilitation from injuries [14]. From a nutrients viewpoint, while carbohydrates (CHO) play an important role in fueling and recovery of endurance athletes [15], adequate protein intake (supplied by supplements or daily diet) is crucial to maintaining muscle protein synthesis, energy production and appropriate immune function [16]. Additionally, numerous metabolic pathways involved in health and physical performance, as well as many physiological mechanisms, are controlled by vitamins and minerals [17], which may not be sufficiently supplied via daily foods in endurance athletes [18]. As a result, the intake of both vitamin and mineral supplements is more prevalent than CHO or protein supplementation among athletes [19, 20]. However, all nutritional supplements categories, including dietary supplements, sports nutrition products and ergogenic supplements, can be an important source of micronutrient intake for athletes should there be deficiencies in their habitual diet [21].

Despite recent developments in theoretical dietary recommendations for endurance athletes [14] and the increased availability of nutritional supplements nowadays [12], nutrition professionals are being challenged to provide practical recommendations for supplement intake. Although endurance athletes are reported to consume more supplements than athletes engaged in sprint and strength activities [22], current evidence-based literature regarding nutrient requirements of distance runners is not consistent and remains an area of debate [5]. For example, the American College of Sports Medicine (ACSM) recommends that endurance athletes should consume between 1.2 and 2.0 g protein per kilogram of their body weight [9]. However, considering the increased protein turnover rate in endurance athletes as well as the important role of protein in fueling, weight maintenance, and immune function, the recommendation is not sufficiently detailed enough for personalized use within all practical and real-world situations. Thus, it appears that there is a fundamental need for a more specified nutritional recommendation based on inter-individual characteristics of different athletic populations. Moreover, different nutritional requirements between elite and recreational endurance athletes [23, 24] highlight the importance of individualized recommendations for nutrient and supplement intake directly based on the athlete’s goal as well as physiological and nutritional demands of a specific athlete. For example, while elite endurance runners seek to promote favourable training adaptations and optimize competition performance, recreational distance runners mainly focus on health aspects of training and/or competition [23, 25]. However, current evidence shows that similar to elite athletes [26], recreational endurance athletes are also at risk of consuming insufficient amounts of some nutrients to meet daily training and health demands [27, 28]. Therefore, recreational athletes may have different or at least limited choices, particularly regarding performance-enhancing supplements and/or those used to cope with environmental challenges (e.g., heat and altitude). When using dietary supplements, however, it is recommended that endurance runners must consider a balance between potential risks (e.g., adverse health effects, distraction, supplement contamination) and benefits (e.g., contribution to health and performance) [23] to meet their higher exercise-induced energy requirements with a maximum level of safety.

Although some athletes consume higher doses of certain micronutrient supplements (e.g. iron, magnesium, vitamin C, and vitamin D, multi-vitamin/mineral) to maximize its beneficial effects [29], it has been well-established that overconsumption can be associated with a diminished state of health and performance particularly with the advancement of time [30]. Additionally, considering the high prevalence of gastrointestinal (GI) disorders among endurance runners [4], caution must be warranted when following high-fat diets, particularly use of medium-chain triglyceride oils [14] or consuming some supplements such as creatine [31] and amino acids [32], which are potentially associated with some GI distress and diarrhea.

According to the limited number and inconsistent scientific reports, the behaviours related to nutrient and supplement intake of recreational and elite endurance athletes are not yet well-understood [20]. Although evidence indicates that long-distance runners use micronutrient (but not macronutrient) supplements more than other track & field disciplines, including shorter-distance runners [19], no study has yet examined the effect of racing distance in endurance runners’ supplement intake. Therefore, the present study aimed to investigate patterns of supplement intake (particularly micro- and macro-nutrient supplements) in female and male recreational runners over the half-marathon, (ultra-)marathon, and 10-km distance. It was hypothesized that regardless of race distance, the majority of runners consume supplements on a regular basis.

## Materials and methods

The methods detailed below have been previously described by Boldt et al. [3], Wirnitzer et al. [33] and Wirnitzer et al. [34].

### Study design and ethical approval

The present study is a part of the NURMI (Nutrition and Running High Mileage) Study Step 2 [33]. The study protocol was approved by the ethics board of St. Gallen, Switzerland (EKSG 14/145; May 6, 2015). The trial registration number ISRCTN73074080.

### Participants and experimental approach

**Fig. 1:**
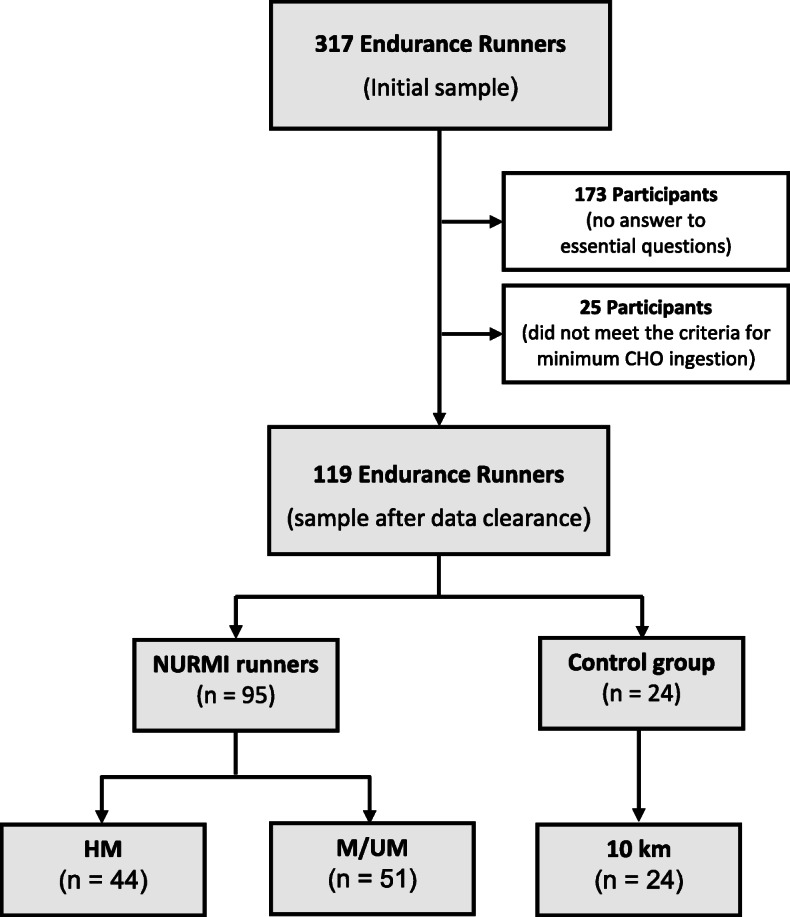
Enrollment and categorization of participants. Race distances: 10 km – 10-kilometers, HM – half-marathon, M/UM – marathon/ultra-marathon.

Endurance runners were mainly recruited from Austria, Germany and Switzerland. They were contacted primarily via social media, websites of organizers of marathon events, online running communities, emaillists and runners’ magazines, as well as via other multi-channel recruitment sources, and through personal contacts. Participants were asked to complete an online survey within the NURMI Study Step 2, which was available in German and English [35]. Prior to participation, subjects were given a detailed explanation of the study procedures, after which they were requested to give informed consent to participate in the study. The questionnaire contained several parts, e.g., running, training and racing behaviour, quality of life, food frequency, as well as dietary information, including supplement intake. For successful participation in the study, the following inclusion criteria were required: (1) written informed consent, (2) at least 18 years of age, (3) questionnaire Step 2 completed, (4) completion of at least a half-marathon distance running event in the past two years. Participants were categorized according to race distance (Fig. 1): half-marathon and (ultra-)marathon (data were pooled since the marathon distance is included in an ultra-marathon). The shortest ultra-marathon distance reported was 50 km, the longest distance was 160 km. In addition, a total of 79 motivated runners who had not participated in a half-marathon or marathon but named a 10-kilometers (10 km) race instead provided accurate and useful answers with plenty of high-quality data. To avoid an irreversible loss of these valuable data sets, those who met the inclusion criteria (1) to (3) were included as an additional race distance subgroup. The characteristics of the participants are presented in Table 1.

**Table 1:** Anthropometric and sociodemographic characteristics of participants

		Total *n* = 119	10 km *n* = 24	HM *n* = 44	M/UM *n* = 51
Age (years)		43 (IQR 15.5)	46 (IQR 13.5)	42.5 (IQR 17)	43 (IQR 15.5)
Body Weight (kg)		68.3 (IQR 16)	66.4 (IQR 15.9)	67 (IQR 12.2)	69.5 (IQR 17.1)
Height (m)		1.8 (IQR 0.1)	1.7 (IQR 0.1)	1.8 (IQR 0.1)	1.8 (IQR 0.1)
BMI (kg/m^2^)		22.4 (IQR 3.47)	21.73 (IQR 3.41)	22.3 (IQR 3.42)	22.19 (IQR 3.22)
Sex	Female Male	53 (45 %) 66 (55 %)	16 (67 %) 8 (33 %)	20 (45 %) 24 (55 %)	17 (33 %) 34 (67 %)
Academic Qualification	No Qualification Upper Secondary Education/Technical A Levels or Equivalent University/Higher Degree No Answer	0 (0 %) 42 (35 %) 29 (24 %) 35 (29 %) 13 (11 %)	0 (0 %) 8 (33 %) 10 (42 %) 5 (21 %) 1 (4 %)	0 (0 %) 15 (34 %) 6 (14 %) 15 (34 %) 8 (18 %)	0 (0 %) 19 (37 %) 13 (25 %) 15 (29 %) 4 (8 %)
Marital Status	Divorced/Separated Married/Living with Partner Single	6 (5 %) 85 (71 %) 28 (24 %)	1 (4 %) 16 (67 %) 7 (29 %)	3 (7 %) 30 (68 %) 11 (25 %)	2 (4 %) 39 (76 %) 10 (20 %)
Country of Residence	Austria Germany Switzerland Other Countries	21 (18 %) 85 (71 %) 8 (7 %) 5 (4 %)	4 (17 %) 17 (71 %) 0 (0 %) 3 (12 %)	8 (18 %) 31 (70 %) 4 (9 %) 1 (2 %)	9 (18 %) 37 (73 %) 4 (8 %) 1 (2 %)
Running Experience	Low Medium High	23 (19 %) 77 (65 %) 19 (16 %)	4 (17 %) 15 (62 %) 5 (21 %)	11 (25 %) 24 (55 %) 9 (20 %)	8 (16 %) 38 (75 %) 5 (10 %)
Racing Experience	Low Medium High	70 (59 %) 33 (28 %) 16 (13 %)	17 (71 %) 5 (21 %) 2 (8 %)	28 (64 %) 10 (23 %) 6 (14 %)	25 (49 %) 18 (35 %) 8 (16 %)

*BMI *body mass index; *IQR* interquartile range; *10 km* 10-kilometers; *HM* half-marathon; *M/UM* marathon/ultra-marathon

### Data clearance

To control for measures of (1) running activity (history, motivation, training, racing etc.) and (2) diet, two groups of control questions were included in the survey. As a result, 69 participants were excluded from data analysis. Additionally, 101 participants with no statement considering running training (e.g., training time) were also excluded from data analysis. In order to control for a minimal health status linked to a minimum fitness level, and to further enhance the reliability of data sets, the Body Mass Index (BMI) approach following the World Health Organization (WHO) standards [36, 37] was implemented. With a BMI ≥ 30 kg/m^2^, however, other health-protective and/or weight loss strategies other than running are necessary to safely reduce body weight first. Therefore, 3 participants with a BMI ≥ 30 kg/m^2^ were excluded from data analysis. Of the remaining 144 participants, another 25 runners were recognized for consuming less than 50 % CHO in their total caloric intake, which is lower than the minimum recommended level to maintain health that serves as a basis for exercise performance ([7, 38]: p 618, 39: p 448). These runners were excluded from the analysis to avoid contradictory data in supplement intake [39].

Altogether, from a total number of 317 endurance runners who completed the survey, 119 participants (53 women, 66 men) remained after clearance with a mean age of 43 (IQR 15.5) years. A total of 24 participants were 10-km runners, 44 half-marathon runners, and 51 (ultra-)marathon runners (Fig. 1). While runners over the 10-km distance were the oldest participants, (ultra-)marathoners were found to have the highest absolute values for body weight, and half-marathoners had the highest absolute values of BMI (Table 1).

### Measures

Participants were asked to report their regular supplement intake, in addition to food frequency (unpublished data from our laboratory based on the validated food frequency questionnaire of the “German Health Interview and Examination Survey for Adults (DEGS)” with friendly permission of the Robert Koch Institute, Berlin, Germany) [40, 41]. Self-reported daily calories described macronutrient intake of endurance runners from CHO, protein, and fat. The term supplement was defined as oral products consumed alongside daily diet, in order to meet nutritional requirements and/or performance goals [12, 13]. Supplement intake was described by the following items: regular intake, frequency, kind of supplement (CHO/protein, minerals, and vitamins), brand of supplement (with nutrient that provides the main contribution), amount, and additional substances. The data was linked to age, sex, specific diet, running distance, and experience of running training and racing.

The latent factors “*running experience*” (“age.first.running event”, “age.run”, “age.first.half-marathon”, “age.first.marathon”) and “*racing experience*” (“years.running”, “completed.half-marathon.number”, “completed.marathon.number”) were each operationalized as sum-index and derived by using both pooled items defined by specific items based on manifest variables. Since the running experience (e.g., number of active running years, age of first running event, number of completed races) depends on age, the respective items were operationalized with age (e.g., age-related years of running, age-related number of completed races over half-marathon distance). With this, the respective items (e.g., age-related start of running, completion of first marathon race) were centered by the median and were z-transformed to a new scale by summarizing the respective items (e.g., years of running, number of completed races over specific distances). Based on this, we categorized the values considering the two latent factors, “*running experience*” and “*racing experience*”, as *low* (values below − 1), *medium* (values ranging from − 1 to + 1), and *high* (values higher + 1).

### Statistical analysis

The statistical software R, version 4.0.0 (R Foundation for Statistical Computing, Vienna, Austria), was used to perform all statistical analyses. Exploratory analysis was performed by descriptive statistics (median and interquartile range (IQR), mean and standard deviation (SD)). Significant differences in supplement intake between race distance subgroups and sex, age, running experience and racing experience were calculated using a non-parametric ANOVA. Chi-square test (χ²; nominal scale) was used to examine the association between race distance subgroups and sex, age, and experience in running training and racing. Kruskal-Wallis tests (ordinal and metric scale) were approximated using the t or F distributions or ordinary least squares, standard errors (SE) and R². Logistic regression analysis (95 % confidence interval (95 %-CI)) was used to determine the effect size of the variables (age, sex, running experience, racing experience) on intake from kind of supplements (macronutrients, minerals, vitamins) and was displayed as effect plots (95 % confidence interval (95 %-CI)). The level of statistical significance was set at *p* ≤ 0.05.

## Results

In descriptive characteristics, a significant difference was found between sex and race distance (χ^2^_(2)_ = 7.36, *p* = 0.025), with the majority of 10-km runners female (67 %), while most of the half-marathoners and (ultra-)marathoners were male (55 and 67 %, respectively). No significant association was found between race distance and age, anthropometry (body weight, height, BMI), academic qualification, marital status, country of residence, running or racing experience (*p* > 0.05).

50 % of participants (*n* = 59) reported consuming supplements regularly. While the intake from CHO/protein was 19 %, the intakes from minerals, vitamins, and other supplements were 34 %, 43 and 6 %, respectively. Consumption of CHO/protein supplements was higher among half-marathoners (22 %) compared to 10-km runners (17 %) and (ultra-)marathoners (16 %). It was found that race distance subgroups had close prevalence rates for mineral supplement intake (33 % in 10-km runners, 31 % in half-marathoners and 34 % in (ultra-)marathoners) and varied prevalence rates for vitamin supplement intake (55 % in 10-km runners, 42 % in half-marathoners and 36 % in (ultra-)marathoners). While multivitamins (31 %) and vitamin B12 (28 %) had the most prevalent use in vitamin supplements, magnesium (19 %) was the highest reported mineral supplement by total runners. When comparing race distance subgroups, a more prevalent consumption of vitamin supplements by 10-km runners, or mineral supplements by half-marathoners, was found compared to their counterparts in other distances. Table 2 shows the prevalence of high-frequently used micronutrient supplements including calcium, iron, magnesium, zinc, multi-vitamin, vitamin B-complex, vitamin B_12_, vitamin C, and vitamin D.

**Table 2:** Prevalence of most frequently used micronutrient supplements

		Total (n = 84)	10-km (n = 19)	HM (n = 31)	M/UM (n = 34)
Minerals	Calcium	5 %	5 %	6 %	3 %
	Iron	7 %	-	10 %	9 %
	Magnesium	19 %	16 %	26 %	15 %
	Zinc	10 %	11 %	10 %	9 %
Vitamins	Vitamin B_12_	28 %	47 %	19 %	26 %
	Vitamin C	7 %	16 %	3 %	6 %
	Vitamin D	14 %	26 %	19 %	3 %
	Vitamin B-complex	5 %	5 %	3 %	6 %
	Multivitamin	31 %	26 %	35 %	29 %

*10 km* 10-kilometers; *HM* half-marathon; *M/UM* marathon/ultra-marathon

Regarding the frequency of supplement intake, 59 % of runners reported consuming supplements regularly with their daily diet, 8 % five or six times a week, 10 % three or four times a week, 17 % once or twice a week and 5 % of participants indicated less than once per week. When comparing dietary groups, 61 % of omnivores, 36 % of vegetarians and 62 % of vegans reported consuming supplements daily. The analysis of the self-reported distribution of macronutrients showed that 58.8 %, 24.0 and 17.2 % of the total energy intake of participants were from CHO, protein and fat, respectively.

**Fig. 2:**
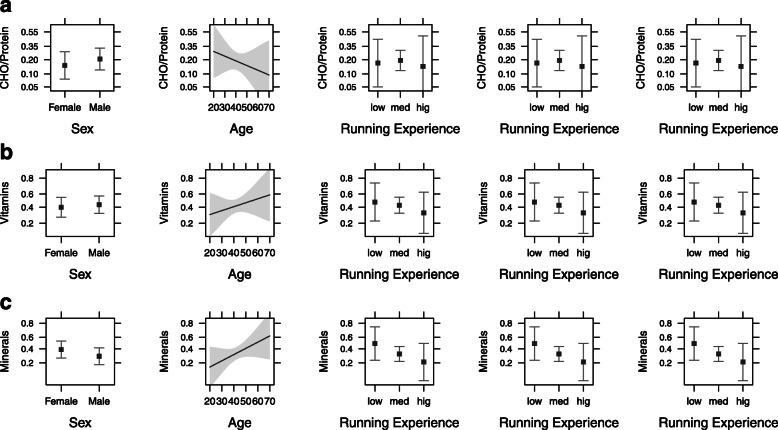
Effect plots with 95 % CI for interactions between sex, age, race distance, running experience, racing experience and supplement intake from carbohydrates/protein (**a**), vitamins (**b**), and minerals (**c**). CHO – carbohydrates. 10 km – 10-kilometers. HM – half-marathon. M /UM – marathon/ultra-marathon

Based on the results from ANOVA and the logistic regression analysis, no significant effect was detected for CHO/protein (χ^2^_(2)_ = 0.60, *p* = 0.742), minerals (χ^2^_(2)_ = 0.10, *p* = 0.954) and vitamins (χ^2^_(2)_ = 1.94, *p* = 0.380) across race distance subgroups (Table 3). There was no significant association between supplement intake and sex, age, running experience (low, medium and high) and racing experience (low, medium and high) (*p* > 0.05) (Fig. 2). No significant effects considering macronutrient intake were observed by sex, age, running experience or racing experience among race distance subgroups (*p* > 0.05).

**Table 3:** Effects of sex, age, race distance, running experience and racing experience on supplement intake

		CHO/Protein			Minerals			Vitamins		
		Fit	CI	***p***	Fit	CI	***p***	Fit	CI	***p***
Sex	**Female**	0.15	[0.08, 0.28]	0.468	0.39	[0.26, 0.53]	0.280	0.40	[0.27, 0.55]	0.719
	**Male**	0.21	[0.12, 0.33]		0.28	[0.18, 0.42]		0.44	[0.31, 0.57]	
Age (y)	**20**	0.29	[0.08, 0.65]	0.424	0.16	[0.04, 0.43]	0.140	0.30	[0.10, 0.62]	0.402
	**30**	0.23	[0.11, 0.43]		0.22	[0.11, 0.41]		0.35	[0.19, 0.55]	
	**40**	0.19	[0.12, 0.28]		0.31	[0.22, 0.41]		0.41	[0.31, 0.51]	
	**50**	0.15	[0.08, 0.27]		0.41	[0.28, 0.55]		0.47	[0.33, 0.61]	
	**70**	0.09	[0.01, 0.43]		0.62	[0.24, 0.89]		0.59	[0.22, 0.88]	
Race Distance	**10 km**	0.17	[0.06, 0.38]	0.742	0.33	[0.16, 0.56]	0.954	0.55	[0.34, 0.75]	0.380
	**HM**	0.22	[0.12, 0.37]		0.31	[0.19, 0.47]		0.42	[0.28, 0.58]	
	**M/UM**	0.16	[0.05, 0.45]		0.34	[0.22, 0.49]		0.36	[0.23, 0.51]	
Running Experience	**Low**	0.17	[0.12, 0.29]	0.901	0.49	[0.23, 0.76]	0.451	0.48	[0.22, 0.75]	0.776
	**Medium**	0.19	[0.12, 0.30]		0.32	[0.22, 0.44]		0.43	[0.32, 0.55]	
	**High**	0.15	[0.03, 0.49]		0.21	[0.07, 0.49]		0.32	[0.12, 0.63]	
Racing Experience	**Low**	0.15	[0.08, 0.27]	0.686	0.27	[0.17, 0.40]	0.195	0.36	[0.24, 0.49]	0.230
	**Medium**	0.21	[0.10, 0.40]		0.36	[0.21, 0.54]		0.46	[0.29, 0.65]	
	**High**	0.25	[0.08, 0.54]		0.55	[0.28, 0.79]		0.63	[0.35, 0.84]	

*CHO* carbohydrates. *10 km* 10-kilometers; *HM* half-marathon; *M/UM* marathon/ultra-marathon; *CI* mean effect size with 95%-CI, upper and lower boundaries; *p p*-value

## Discussion

The present study aimed at investigating supplement intake behaviours related to sex, age, running experience and racing experience in recreational endurance runners competing over different race distances (10-km, HM, M/UM).

The most important findings were (i) 50 % of the distance runners were found to consume supplements regularly, (ii) vitamin supplements had the highest intake rate in runners by 43 % compared to minerals (34 %) or CHO/protein supplements (19 %), (iii) no differences between race distance groups were detected regarding supplement intake from CHO/protein, minerals or vitamins, (iv) age, sex, running and racing experience showed no significant effect on supplement intake and (v) 59 % of runners reported to consume supplements with their daily diet as the most prevalent frequency of supplement intake.

### Race distance and BMI

Generally, endurance runners had a lower BMI than normal populations [42]. It has been shown that running performance is significantly associated with BMI [43, 44] with a parabolic function between running speed and BMI [45]. In the present study, no significant differences were observed between HM, M/UM and 10-km runners with a mean BMI of 22.30, 22.19 and 21.73 kg/m^2^, respectively. Compared to the present findings, both higher [46] and lower [47] values of BMI have been previously reported in distance runners. However, considering the elimination of obese participants from the present study (by applying the exclusion criteria for runners with BMI > 30), caution must be considered when comparing the present sample with similar studies. Although evidence indicates that a lower BMI and smaller body size are associated with improved endurance running performance [43, 48, 49], some studies exclude marathon runners or ultra-endurance athletes from this evidence [44, 50], which is consistent with the present findings where the M/UM runners had a slightly but non-significantly higher BMI than the 10-km runners. In general, the higher BMI of ultra-marathon runners compared to short-distance endurance runners is possibly due to the advanced importance of energy stores than running speed in high-distance activities [51].

### Race distance and supplement intake

Comprehensive characteristics of supplements and the scenarios in which they contribute to nutritional requirements of endurance athletes were recently presented by Burke et al. [6] and Maughan et al. [13]. In general, performance improvements and meeting nutrient requirements to optimize training demands and recovery are two major goals that persuade endurance runners to use supplements [6, 13]. In the present study, one out of two endurance runners consumed supplements regularly, which is lower compared to previous reports by other European [20, 52, 53], Canadian [54] and Japanese [19] studies on elite athletes. In this regard, a possible justification is the fact that, unlike other studies, the majority of participants in the present study were recreational runners, who have been shown to use supplements less frequently than elite athletes [55, 56]. A comprehensive study performed on 10,274 athletes observed vitamin/mineral supplement intake in 59 % of elite athletes [57]. Nevertheless, a wide range for the prevalence of supplement use has been reported in a meta-analysis, and due to the lack of homogeneity in the available studies, the authors were unable to identify a clear, conclusive prevalence of supplement intake in athletes [55]. However, it is well-established that endurance athletes use supplements to a greater extent than athletes from other sport categories such as team [58] or power-based [19, 22, 54] sports.

Although there was no significant difference in CHO/protein, mineral or vitamin supplementation between half-marathoners, (ultra-)marathoners and 10-km runners, to the best of the authors’ knowledge, no study exists comparing different groups of distance runners and patterns of supplement intake. Another important finding of this study was that 59 % of runners reported a daily intake of supplements, which is 12 % higher compared to the findings from a recent study on elite athletes from 15 different kinds of sports [52]. This contradictory evidence is perhaps due to different guidelines for supplement consumption in different sports [13].

Despite substantially higher exercise-induced nutrient requirements associated with long-distance competition, there is insufficient evidence that intake of multi-vitamin and/or mineral supplements leads to advantageous effects in marathoners, except for the case of a clinically determined nutrient deficiency [5]. The endurance runners in the present study most frequently consume supplements for the supply of vitamins (43 %), but minerals (34 %) and CHO/proteins (19 %) supplementation was less pronounced, which is consistent with previous reports indicating multi-vitamins as the supplement with the highest prevalence consumed by athletes [20, 29], particularly long-distance runners [19]. This finding might be associated with the lower micronutrient density of routinely mixed diets of endurance athletes [18, 20]. However, the different nutritional needs in different sports [55] has caused the emergence of contradictory evidence reporting creatine [59], amino acids [60] or proteins [52, 55] as the most frequently consumed supplements by athletes in general. Interestingly, a study performed on ultra-marathon runners who consumed mineral or vitamin supplements four weeks before a race found that ultra-endurance athletes finished the competition not faster than athletes with no pre-race intake of minerals and vitamins [61]. From a practical point of view, however, endurance runners’ nutritional strategies, including supplement intake, should be personalized depending on the training status, basal metabolic rate, daily energy expenditure, thermic effect of food, specific training requirements, body composition goals and environmental conditions as well as frequency, intensity and duration of training and racing sessions [46].

In the present study, analysis of runners’ open comments showed that despite meeting reference values for micronutrient intake, the consumption prevalence of many micronutrient supplements seems to vary between different race distance subgroups. Amongst runners, 10-km runners had a higher tendency to consume vitamin supplements, while half-marathoners reported to consume mineral supplements more than 10-km and (ultra-)marathon runners. In addition, various micronutrients, macronutrients and performance-enhancement substances (e.g., caffeine and nitrate), separately or in conjugation with other nutrients, were stated by runners in open comments but without further details to be expressively summarized. This finding was due to the nature of open questions in which runners provided inconsistent answers, missing information or double answers. Therefore, as previously mentioned by another study on marathoners [62], caution is advised when interpreting the estimated frequencies of supplement intake due to the varied nature of supplement products, which mostly contain secondary ingredients.

### Sex differences

In line with other studies which have focused on sex differences in patterns of dietary supplement intake, indicating a similar prevalence of use in male and female athletes [55, 59, 63], there were no significant effects of sex on the type of supplement intake found in the present study. This finding might be possibly due to significant sex differences in race distance subgroups in the present study. Contrary to this, and although the participants’ mean age is markedly lower than the present study (18 vs. 43 years), a study of 32 track and field athletes competing at the World Junior Championships 2004 [64] found that 62 % of athletes consumed supplements with a markedly higher intake in females (75 %) than males (55 %), which is in line with another study also reporting a 20 % difference in supplement intake between male (32 %) and female (52 %) athletes [65]. However, the evidence is inconsistent, which indicates a significantly greater supplement intake prevalence in males than females [60]. Interestingly, evidence indicates a larger proportion of female athletes use mineral and vitamin supplements, whereas most male athletes use protein and ergogenic supplement [59], as well as creatine [55]. This finding supports other studies in which males and females were asked for their reasons to use dietary supplements, where males reported strength gaining and muscle mass as a higher priority than health and/or meeting dietary guidelines, while females mentioned health as their first priority [54, 66]. Moreover, the higher consumption of protein supplements by male athletes can also be explained by recent finding from the NURMI Study (unpublished data from our laboratory), which indicates a higher training volume in male distance runners compared to their female counterparts [67].

### Age differences

The prevalence of supplement intake appears to be endemic in all age categories of the athletic population, and evidence reports that supplement use among athletes begins at an early age [56]. The majority of studies investigating age differences in nutrient or supplement intake have compared young vs. adult athletes [68, 69]. In the present study, we have a wide range of ages from 20 to 70, allowing for an improved understanding of age-related differences in supplement intake. In line with the present findings, another study showed that there is no association between age and supplement intake [60]. This finding is inconsistent with the majority of previous investigations on patterns of supplement use among athletes, where prevalence and number of supplements consumed were shown to rise with increasing age [19, 69–71], which might be explained by the lower level of professionalism in junior athletes compared to the increased expertise of senior athletes [56]. On the contrary, another study showed that younger athletes had a higher intake of both sport nutrition products and dietary supplements compared to older athletes, and the researchers mentioned current marketing strategies that appeal to younger populations as the possible cause [53]. Thus, the aforementioned contradictory findings show that the age-related effects of supplement intake in athletes remain unclear, and it seems to be highly dependent on the level of professionalism. This result might be consistently in line with the age-related findings in the present study, where the majority of participants were recreational runners.

### Differences in running and racing experiences

While several studies have investigated the influence of distance runners’ racing and/or running experience on different variables including, but not limited to, running mechanics [72], running injuries [73], training characteristic [74], hydration practices and perceptions [75] and medical complications [76], to the best of the authors’ knowledge, to date no study has been conducted examining the association between running or racing experience on supplement intake in endurance athletes. In the present study, neither running experience nor racing experience was associated with the type of supplements taken by distance runners. If the close connection between running experience and performance level are considered, the present finding seems to be inconsistent with the fact that elite athletes use dietary supplements much more than their non-elite counterparts [55, 56]. It is generally accepted that dietary strategies at the elite and professional level are necessary to cope with their higher nutritional needs and to ease the GI tract in order to limit GI complications that might negatively affect in-race performance, refuelling, and recovery. In this regard, Braun et al. [68] showed that the age-associated differences in supplement intake appear to be connected to the level of performance. Training volume also appears to be associated with supplement intake, as athletes training at a high volume reported a 20 % greater prevalence of supplement intake compared to athletes with a lower training volume [60].

### Macronutrient distribution

From a metabolic perspective, runners over long distances and at lower intensities rely heavily on aerobic metabolism to efficiently utilize their muscle and liver glycogen and body fat stores [77]. Therefore, careful consideration of the macronutrient requirements of both training and recovery is recommended to achieve nutritional balance in order to adequately match the higher exercise-induced needs of distance runners [78]. According to well-established guidelines for macronutrient intake, to sufficiently supply for higher nutritional requirements of endurance athletes, macronutrient distribution of at least 60 % from carbohydrates (5, 39: p 448, [79]), 10–15 % from protein ([5, 80]: p 471) and 25–30 % from fat [5] is recommended. Compared to the current guidelines, and irrespective of racing distance, the self-reported estimation of total daily calorie intake was 58.8 % from CHO, which is close to matching the recommended intakes while daily protein intake (24.0 %) was reported higher, and fat intake was reported markedly lower (17.2 %). Regardless of the fact that the present values of macronutrient intake are influenced by one of the implemented exclusion criteria in this study (CHO intake by 50 %), two possible reasons seem to contribute to explain present findings. Firstly, distance runners are frequently recommended to consume adequate protein by nutritional guidelines, suggesting between 1.2 and 2.0 g/kg body weight [9, 21], which is 1.5–2.5 times greater than non-athlete populations [9]. Secondly, since our data is based on a self-reported estimation, probable misreporting of dietary protein (over-reporting) and fat (under-reporting) might have led to socially desirable statements of self-estimation, which is somewhat prevalent in the athletic population [81]. In the present study, macronutrient distribution (in terms of self-reported percentage of daily calories) was not only similar between race distance subgroups but also not significantly associated with age, sex, running experience or racing experience. This might be a result of similar physiological demands between shorter- and longer-distance runners [82].

### Limitations

In addition to the relatively small sample size (initially n = 317; finally enrolled n = 119), this study shares with others the limitations of the cross-sectional design. The fact that the findings relied on self-reported data should be considered further as the primary limitation since under- and over-reporting are potentially prevalent in self-reported records. Self-reports for this type of variable are valid if they are collected immediately or shortly after an event [83]. In this study, however, the average time between completion of the last event and completion of the questionnaire by the participants was not known (see inclusion criteria: self-reports refer to at least one running event completed within the past two years). Therefore, the reliability of the data depends on the conscientiousness of the runners, and thus caution is advised when inferring based on the concluded associations. However, in order to minimize this effect, control questions were implemented in different parts of the questionnaire, and the participants’ statements were checked for congruency and meaningfulness. Moreover, the potential selection bias may have affected the present results as (i) the majority of the participants (96 %) were from German-speaking countries, and (ii) 1 in 2 participants in the present study (52 %) stated following a vegan or vegetarian diet (likely with specific dietary patterns), which is markedly higher than in other Western nations and particularly German-speaking countries (10–14 %). Another limitation of the present study could be the impossibility to conduct statistical analysis for inconsistent and incomplete open comments provided by runners, which compelled the authors to report the relevant findings inclusively. Furthermore, the survey used in this study did not provide information regarding training or racing phases, however, the supplement intake portion of the survey asked participants to report based off their preparation and competition seasons.

Despite the above-mentioned limitations, the present findings deliver valuable and novel information and thus add to the existing body of scientific evidence about dietary supplement intake with a special focus on endurance runners coping for different race distances. In addition to opening up a direction for future clinical trials, the results from the present study may provide a window into the targeted approaches of distance runners, as well as their coaches and sport nutrition specialists, considering sustainable nutritional strategies for long-term training and competition adherence. Future research with large randomized samples of distance runners will assist in providing comparable data on patterns of supplement intakes in order to support and help to meet the guidelines for macronutrient, vitamin and mineral intakes, especially for better understanding the use of supplement intake in recreational distance runners over 10-km, HM and M/UM distances.

## Conclusions

In summary, this study shows that 50 % of distance runners were found to regularly consume micro- and macro-nutrient supplements, with 59 % consuming supplements on a daily basis. Vitamin supplements had the highest intake rate by 43 % compared to minerals (34 %) and CHO/protein supplements (19 %). Type of supplement intake did not have any association with race distance (from 10-km to ultra-marathon), age, sex, running experience or racing experience. Considering health and sports nutrition counselling for endurance runners, the lack of difference in patterns of supplement intake between runners of different race distance subgroups may sufficiently indicate an emphasis on optimizing personalized nutritional strategies independently of training and race distance. It seems practicable for recreational endurance runners to consider their running-induced supplemental intake as a part of the everyday dietary pattern; therefore, we suggest optimizing these habits by receiving consultations from specialized sports nutritionists. Sport supplement producers/manufacturers could also take into account the patterns of supplement intake amongst recreational runners in their production policies, as running is a worldwide mass sport with millions of active runners across the globe.

## Data Availability

The datasets generated during and/or analyzed during the current study are not publicly available, but may be made available upon reasonable request. Subjects will receive a brief summary of the results of the NURMI Study if desired.

## Abbreviations

NURMI: Nutrition and Running High Mileage
HM: Half-marathon
M/UM: Marathon/Ultra-marathon
ACSM: American College of Sports Medicine
GI: Gastrointestinal
WHO: World Health Organization
ANOVA: Analysis of Variance
BMI: Body Mass Index
DEGS: German Health Interview and Examination Survey for Adults
IQR: Interquartile Range
SD: Standard Deviation
UK: United Kingdom
CHO: Carbohydrate
DACH: Germany, Austria, and Switzerland
