# Correction to: Supplement intake in half-marathon, (ultra-)marathon and 10-km runners – results from the NURMI study (Step 2)

**DOI:** 10.1186/s12970-021-00473-x

**Published:** 2021-12-24

**Authors:** Katharina Wirnitzer, Mohamad Motevalli, Derrick Tanous, Martina Gregori, Gerold Wirnitzer, Claus Leitzmann, Lee Hill, Thomas Rosemann, Beat Knechtle

**Affiliations:** 1 Department of Subject Didactics and Educational Research and Development, University College of Teacher Education Tyrol, Innsbruck, Austria; 2 Department of Sport Science, University of Innsbruck, Innsbruck, Austria; 3 Life and Health Science Cluster Tirol, Subcluster Health/Medicine/Psychology, Innsbruck, Austria; 4 Research Center Medical Humanities, Leopold-Franzens University of Innsbruck, Innsbruck, Austria; 5 Faculty of Physical Education and Sports Sciences, Ferdowsi University of Mashhad, Mashhad, Iran; 6 Department of Nutritional Sciences, University of Vienna, Vienna, Austria; 7 AdventureV & Change2V, Stans, Austria; 8 Institute of Nutrition, University of Gießen, Gießen, Germany; 9 Department of Pediatrics, Divison of Gastroenterology and Nutrition, McMaster University, Hamilton, Canada; 10 Institute of Primary Care, University of Zurich, Zurich, Switzerland; 11 Medbase St. Gallen Am Vadianplatz, St. Gallen, Switzerland

**Correction to: J Int Soc Sports Nutr. 18, 64 (2021)**

**https://doi.org/10.1186/s12970-021-00460-2**

The original article [1] mistakenly inverted each author’s name in the authorship. This has since been corrected.
